# The impact of quality on hospital choice. Which information affects patients’ behavior for colorectal resection or knee replacement?

**DOI:** 10.1007/s10729-020-09540-2

**Published:** 2021-01-27

**Authors:** David Kuklinski, Justus Vogel, Alexander Geissler

**Affiliations:** 1 Department of Health Care Management, Technische Universität Berlin, Strasse des 17. Juni 135, 10623 Berlin, Germany; 2 School of Medicine, University of St. Gallen, St. Jakob-Strasse 21, 9000 St. Gallen, Switzerland

**Keywords:** Health policy, Provider choice, Quality of care, Quality transparency, Competition, C25, D82, D90, H51, I11, I18

## Abstract

Quality competition among hospitals, induced by patients freely choosing their hospital in a price regulated market, can only be realized if quality differences between hospitals are transparent, understandable, and thus influence patients’ hospital choice. We use data from ~145,000 German patients and ~ 900 hospitals for colorectal resections and knee replacements to investigate whether patients value quality and specialization when choosing their hospital. Using a random utility choice model, we estimate patients’ marginal utilities, willingness to travel and change in hospital demand for quality improvements. Patients respond to service quality and specialization and thus, quality competition seems to be present. Colorectal resection patients are willing to travel longer for more specialized hospitals (+9% for procedure volume, +9% for certification). Knee replacement patients travel longer for hospitals with better service quality (+6%) and higher procedure volume (+12%). However, clinical quality indicators, often difficult to access and interpret, barely play a role in patients’ hospital choice. Furthermore, we find that competition on quality for colorectal resection is rather local, whereas for knee replacement we observe regional competition patterns.

**Supplementary Information:**

The online version contains supplementary material available at 10.1007/s10729-020-09540-2.

## Highlights

- We merge data from several sources and include 66,645 colorectal resection patients treated in 862 hospitals and 82,015 knee replacement patients treated in 844 hospitals in Germany into our sample
- Using a utility choice model, we found that patients are willing to travel between 1 and 12% longer for better quality and more specialized hospitals, in which specialization is more important for colorectal resection and service quality for knee replacement
- Consequently, by improving quality and specializing, hospitals can increase their demand by 2 to 28%
- Clinical quality indicators are less available, quite complex and barely relevant for patients, - policy makers therefore need to aim at developing new clinical quality indicators that are more intuitive and patient-oriented
- Hospital management can tailor its their strategic approach to patients’ preferences for respective treatments according to the findings of our study

## Introduction

In Germany, patients can freely choose the hospital they feel is most suitable for their treatment [1, 2]. Simultaneously, hospitals are required to act as independent economic units [1]. As prices are fixed, hospitals can only maximize their revenue by increasing case volume. Especially in areas with overcapacities, hospitals need to find ways to distinguish themselves from competitors in order to attract patients [1]. One opportunity to gain a competitive edge is to influence patients’ hospital choice by offering better quality, thus leading to a competition for patients through quality [3].

Several studies show that free hospital choice and the consequent quality competition ultimately lead to better hospital outcomes [3–5]. Nevertheless, quality competition will only be fully effective and incentivized by hospital management if patients care about quality variation and use it in their decision-making process [6]. Several preconditions must be met: Firstly, quality information is transparent and accessible to patients and referring outpatient physicians. Secondly, quality information is prepared in an understandable and interpretable way. Thirdly, each additional quality metric adds new and incremental information to already existing quality metrics [7]. Fourthly, patients act rationally, i.e. they prefer better quality to worse quality [8]. Lastly, patients must be able to choose from a diverse hospital set in terms of quality. Thus, a certain variance in quality between hospitals must be present [9]. Indeed several studies show that quality differences prevail across hospitals for several treatment areas and geographies [10–13].

In this study, we aim to answer the following questions: First, do patients include quality information into their decision-making process? Second, if they do, which kind of quality matters most? Third, does accessibility and/or comprehensibility of quality information affect the impact of different quality indicators? And fourth, will the impact of quality information differ for “life-saving” treatments such as colorectal resections compared to “life-improving” treatments such as knee replacements?

Up until now, patients’ hospital choice with respect to quality has been examined foremost in the United States and the United Kingdom. Just a few studies can be found for selected European countries such as Germany, Italy and the Netherlands. Studies usually explore elective procedures in orthopedic and cardiologic settings. Several studies investigate objective clinical outcome indicators such as mortality, revision, readmission and re-operation rates; the selection of indicators being based on the procedure and data availability [5, 14–18]. Additionally, literature on patients’ hospital choice covers the impact of subjective quality indicators such as hospital reputation based on patient experience questionnaires [17, 19, 20] or on hospital reputation according to physicians’ opinions [9]. With the emergence of patient-reported outcome measures (PROMs) in recent years, first studies examine the influence of changes of PROM scores on hospital choice [21]. In a systematic review of patients’ hospital choice literature, Brekke et al. [3] found that most studies report a positive impact of quality improvement on patients’ hospital choice and hospital demand.

Our paper contributes to the existing literature in multiple ways. Firstly, we co-explore two treatment areas that are fundamentally different in their impact on patients. Secondly, only few studies for patient choice exist for Germany. Thirdly, literature on the impact of certifications on patients’ decision-making is scarce. Fourthly, we examine the impact of procedure-specific hospital awards on patients’ hospital choice. Hospital awards are based on physicians’ opinion – thus, we can approximate the effect inpatient and referring outpatient physicians have on patients’ hospital choice. Further, we express the patients’ travel burden in terms of travel time instead of distance covered, which we believe better reflects patients’ disutility from travel. Lastly, we include a variety of quality information covering clinical quality indicators, patient recommendation, specialization information (procedure volumes, certifications), physicians’ recommendation (top hospital awards) and structural information into one single model. We further categorize all quality indicators by their degree of accessibility and comprehensibility for patients. We expect that quality indicators with a higher degree of accessibility and comprehensibility have a larger effect on patients’ hospital choice, as suggested by other recent studies [19–21].

To model patients’ hospital choice, we use a random utility choice model. By controlling for observable patient characteristics, we can calculate the utility of a reference patient. In a second step, we estimate the marginal utility of each quality indicator. Lastly, we calculate the willingness to travel and changes in hospital demand for improvements in quality and specialization. As input for the model we use hospital and patient-level data from seven different domains. A detailed description of the data set follows in section 2. Section 3 lays out the econometric model. Section 4 presents model results. Section 5 discusses results and limitations and concludes.

## Data

Patients receiving colorectal resection or knee replacement were identified by procedure codes according to the classification of the German Society for General and Visceral Surgery or the classification of the Joint Federal Committee (see Tables A1 and A2 in the supplementary material). Further, approximately 61% of all patients receiving colorectal resections are diagnosed with cancer. The remainder are also predominantly treated due to an otherwise fatal condition.

### Data sources, levels (patient or hospital) and observed variables

**Fig. 1:**
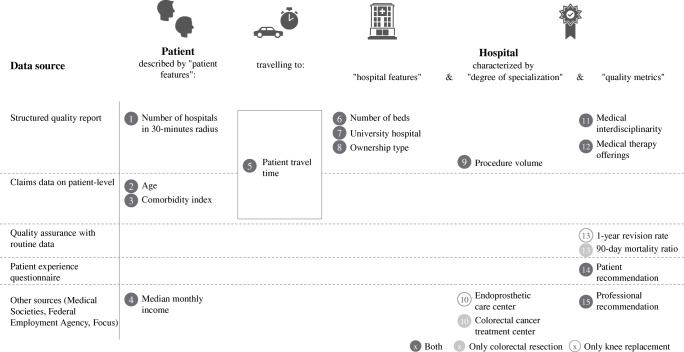
Variables and data sources

Figure 1 gives an overview of the data sources and levels and the observed variables. Patients described by patient features (1–4) travel in a certain time (5) to their hospital of choice. Each hospital is described by hospital features (6–8), indicators for specialization (9–10) and quality metrics (11–15). A detailed description of the used data sources is provided in Appendix A1 in the supplementary material and other recent studies [13, 19, 22].

Claims data describing patients was made available by the largest German health fund AOK (*Allgemeine Ortskrankenkasse*). The patient sample comprises approximately 35% (33%) of the total procedure volume for knee replacement and 33% (35%) of the total procedure volume for colorectal resection in 2017 (2018). We retrieved information on age (2) and the number of comorbidities from the provided claims data. To adjust for comorbidities, we calculated each patient’s Charlson Comorbidity Index [23], shown in Fig. 1 (3). Further, we linked the monthly median income (4) on county level provided by the German Federal Employment Agency [24] to each patient using patients’ zip codes provided in the claims data. Lastly, to control for the variety of choices a patient possesses in proximity, we also described each patient by the number of hospitals within a 30-min radius around the patient’s home (1).

Travel time (5) is defined as the time a patient needs to drive with a standard passenger car and under normal traffic conditions from the centroid of the home zip code area to the coordinates of the hospital. Coordinates of all hospitals and zip code area centroids were obtained by crawling Google API. To calculate driving times between each of the patient - hospital combinations, we used the Stata command *osrmtime* [25] and a local Open Source Routing Machine server [cf. 19].

Hospitals are described by a list of general features, indicators for specialization and quality metrics. Hospital features comprise number of beds, university hospital status and ownership type. Procedure volume (9) and certifications (10) are indicators for the degree of a hospital’s specialization. Most information (6–9) could be retrieved from annual structured quality reports. We collected certification information for colorectal cancer treatment center^1^ from the German Cancer Society^2^ and the certification information for endoprosthetic care center from EndoCert.^3^

As quality metrics, we consider five indicators per procedure. Firstly, we include the patient recommendation score^4^ (14) for each hospital from the patient experience questionnaire data. This score serves as an overall proxy for patient-reported hospital service quality, where a score of 1 specifies the best possible and a score of 6 the worst possible service quality. Secondly, for both procedures we include two variables describing procedure-specific structural quality, namely medical interdisciplinarity^5^ (11) and medical therapy offerings^6^ (12) available in the structured quality reports. Thirdly, as clinical quality indicators, we included the 90-day mortality ratio (13) for colorectal resection and the 1-year revision rate (13) for knee replacement. These indicators are taken from the quality assurance with routine data program (QSR) of the AOK. Lastly, we include professional recommendation (15) as a quality metric. We consider a hospital to be recommended by professionals if it received a hospital award by the German magazine *Focus*. Hospital awards are based on hospital recommendations from inpatient physicians and structured interviews with referring outpatient physicians and specialists. We consider the orthopedic department award for knee replacement and the award for colorectal cancer treatment for colorectal resection.

Quality metrics (except professional recommendation) and indicators for specialization (except certification) are included with a two-year time lag as this information is published with a two-year delay^7^ [22]. Regarding certification, all certifications valid until the end of each year of observation were considered.

Our final sample consists of 66,645 (82,014) patients treated in 862 (844) hospitals for colorectal resection (for knee replacement) in 2017 and 2018. In order to minimize statistical noise and manage computational power needs of the model we performed several data cleansing steps. A detailed description can be found in Appendix A2 in the supplementary material.

### Accessibility and comprehensibility

Despite a generally high degree of quality transparency in Germany [22], the degree of transparency among different quality indicators differs greatly. To discuss the influence of our quality indicators on patient’s hospital choice, we categorized indicators according to their accessibility and comprehensibility. Accessibility is defined by “how accessible the respective quality indicator is to the patient” and assessed on a scale from “not at all” to “easily accessible on trusted public platforms”. Comprehensibility is defined by “how easily a medical layman is able to understand and interpret the quality information” and is evaluated from “only with physician’s help” to “easily understandable”. A detailed overview can be found in Fig. 6 in the supplementary material.

Patient recommendation, procedure volume and structural quality indicators are prepared transparently on frequently visited online platforms^8^ [26]. Certifications and professional recommendation are prominently displayed on hospital websites. Clinical outcome indicators are either not published at all (colorectal resection) or require a diligent search^9^ on online platforms (knee replacement). Despite 90-day mortality not being published, we are interested in its effect on patients’ hospital choice. Studies have shown that patients value higher hospital quality even before quality information becomes available [27, 28]. In contrast, other authors find that clinical quality indicators’ effect on patients’ hospital choice is insignificant [21].

Regarding comprehensibility, we consider recommendation scores and professional recommendation to rank high, and procedure volume and certifications to follow closely. Clinical outcome indicators, on the contrary, are rather difficult to interpret for medical laypersons. Nevertheless, as patients often rely on their referring outpatient physician’s professional opinion [29] and outpatient physicians strongly value high clinical quality [30], we expect some effects from better clinical outcome to affect patient’s hospital choice.

## Econometric model

### Model specification

We are foremost interested in whether patients favor high-quality hospitals over low-quality hospitals and how selected quality indicators affect patients’ utility. We use a random utility choice model [31] to estimate the utility a patient *i* = 1, …, *I* receives from choosing hospital *j* = 1, …, *J* at time *t* = 1, …, *T* where a patient’s utility can be described by observable utility *V*_*ijt*_ and unobserved random utility *v*_*ijt*_.

1 $$ {U}_{ij t}={V}_{ij t}+{v}_{ij t}={\beta}_{tt,i}{TT}_{ij}+{\beta}_{tt^{\mathbf{2}},i}{TT}_{ij}^{\mathbf{2}}+{\beta}_{tt^{\mathbf{3}},i}{TT}_{ij}^{\mathbf{3}}+{\beta}_{q,i}{Q}_{j,t-\mathbf{2}}^{\prime }+{\beta}_{c,i}{C}_{j,t}^{\prime }+{\beta}_{pv,i}{PV}_{j,t-\mathbf{2}}+{\beta}_{a,i}{A}_{j,t}+{\beta}_{z,i}{Z}_j^{\prime }+{v}_{ij t} $$

Observable utility depends on patient travel time *TT*_*ij*_, two-year time-lagged hospital quality metrics ${Q}_{j,t-2}^{\prime }$, certifications ${C}_{j,t}^{\prime }$, two-year time-lagged procedure volume *PV*_*j*, *t* − 2_, professional recommendation *A*_*j*, *t*_, and finally on time-invariant hospital features ${Z}_j^{\prime }$. Patients choose from a predefined choice set *M*_*it*_ comprising the 50 hospitals closest to their home**.** We assume that *v*_*ijt*_ are independent and identically distributed and therefore employ a multinomial logit model (MNL) in which a patient *i* chooses hospital *j* at time *t* with probability *P*_*ijt*_:

2 $$ {P}_{ijt}=\frac{e^{V_{ijt}}}{\sum_{j^{\prime}\in {M}_{it}}{e}^{V_{i{j}^{\prime }t}}} $$

We expect a non-linear effect of travel time on patient’s utility [16, 19, 21]. Disutility is expected to rise quickly for short travel times but diminishes in speed for long travel times and increases again for very long travel times.

Furthermore, patient preferences are allowed to vary with observed patient features including age, comorbidity, median income and the number of hospitals in a 30 min radius. Therefore, marginal utility of relevant indicators for patient *i* can be expressed as:

3 $$ {\beta}_{x,i}={\beta}_x+{X}_i^{\prime }{\delta}_x $$

where *x* represents quality indicators, procedure volume, certifications, and time-invariant hospital features. By mean-centering all variables in ${X}_i^{\prime }$, the coefficients *β*_*tt*_, ${\beta}_{tt^2}$, ${\beta}_{tt^3}$, *β*_*q*_, *β*_*c*_, *β*_*pv*_, *β*_*a*_, *β*_*z*_ describe the preference of the reference patient. Our results are estimated in Stata 16 using the commands *clogit* and *cmclogit.*

### Endogeneity

The model’s estimators are unbiased when all explanatory variables are exogenous, meaning that the error term *v*_*ijt*_ is uncorrelated with any of the explaining variables. The most common types of exogeneity violations are simultaneity bias and omitted variable bias.

A simultaneity bias may arise due to volume-outcome relationships for several treatment areas [14, 32]. Studies have found positive effects of procedure volume on outcome quality for colorectal resection for carcinoma and colorectal resection for diverticulosis, two procedures that are part of our procedure definition for colorectal resection as well as for knee replacements [33]. Nevertheless, we eliminated this bias by including a two-year time lag on quality indicators. Demand at time *t* cannot influence quality in *t* − 2.

Simultaneity bias might also arise through discrimination of hospitals by patients or of patients by hospitals. Sicker patients might choose higher quality hospitals or hospitals might prefer patients with fewer comorbidities, affecting clinical outcome indicators. If one of these behaviors proves to be systematic, we must assume that patient behavior influences clinical quality and at the same time clinical quality influences the patient choice. Fortunately, QSR indicators account for this possible bias with adjustment for patient risks [22].

Moreover, unobserved hospital characteristics may affect patients’ utility and simultaneously impact observed covariates [28] causing omitted variable bias. For example, staff levels and qualification might affect hospital quality and patient utility alike. In order to eliminate potential omitted variable bias we estimate the choice model with alternative-specific time-invariant fixed effects (FEs) [21, 34] and compare it to our main model. The FE model only considers the changes in hospital features and quality indicators in the two years of observation. To cancel out the effect of hospital shifts in our sample from one year to the other, we only include hospitals that were present over the complete time span of our data collection.

### Willingness to travel, demand responsiveness, and elasticities

Estimated coefficients of our MNL model express the marginal utilities for the reference patient. Marginal utilities are difficult to interpret, as they only indicate variables’ directive effect on patient utility. The calculation of marginal rates of substitution allows us to interpret the magnitude of their effect. A commonly-used marginal rate of substitution in the patient choice literature is the so-called willingness to travel (WTT)^10^ [16, 19]. WTT for a one standard deviation increase in quality is estimated as (see also [21]):

4a $$ {\displaystyle \begin{array}{c} WTT=\raisebox{1ex}{$\frac{{\partial TT}_{ij}}{{\partial Q}_j}$}\!\left/ \!\raisebox{-1ex}{${U}_{ij}$}\right.\  SD(Q)=\raisebox{1ex}{$-\frac{{\partial U}_{ij}}{{\partial Q}_j}$}\!\left/ \!\raisebox{-1ex}{$\frac{{\partial U}_{ij}}{{\partial TT}_{ij}}$}\right. SD(Q)\\ {}=\frac{-{\beta}_q}{\beta_{tt}+2{\beta}_{tt^2} TT+3{\beta}_{tt^3}{TT}^2} SD(Q)\end{array}} $$

Respectively, for an increase in procedure volume, for being awarded a top hospital in the respective treatment area and for holding a treatment-specific certification as:

4b1 $$ {WTT}_{PV}=\frac{-{\beta}_{pv}}{\beta_{tt}+2{\beta}_{tt^2} TT+3{\beta}_{tt^3}{TT}^2} SD(PV), $$

4c $$ {WTT}_A=\frac{-{\beta}_a}{\beta_{tt}+2{\beta}_{tt^2} TT+3{\beta}_{tt^3}{TT}^2}, $$

4d $$ {WTT}_C=\frac{-{\beta}_c}{\beta_{tt}+2{\beta}_{tt^2} TT+3{\beta}_{tt^3}{TT}^2}. $$

WTT is calculated separately for each procedure. Travel time is averaged over all patients. *SD*(*Q*) and *SD*(*PV*) are calculated based on all hospitals in the sample. WTT for professional recommendation expresses how much longer a patient is willing to travel for a hospital that was decorated with a hospital award compared to not being decorated. The same logic applies to the WTT for holding a certification.

Moreover, we are interested in estimating the demand change with respect to changes in the aforementioned indicators [21, 35]. Expected demand for hospital *j* can be written as the sum of probabilities of each patient in our data set to choose hospital *j* if *j* is part of her choice set (*i* ∈ *S*_*jt*_):

5 $$ {Y}_{jt}={\sum}_{i\in {S}_{jt}}{P}_{ijt} $$

We can then express the demand responsiveness to changes in e.g., quality as:

6 $$ \frac{{\partial Y}_{jt}}{{\partial Q}_{j,t-2}} SD(Q)= SD(Q){\sum}_{i\in {S}_{jt}}\frac{{\partial P}_{ijt}}{{\partial Q}_{j,t-2}}= SD(Q){\sum}_{i\in {S}_{jt}}{\beta}_q{P}_{ijt}\left(1-{P}_{ijt}\right) $$

and average it over all hospitals in the data set. Demand responsiveness with respect to change in procedure volume, being awarded top hospital and holding a certification is expressed accordingly. Furthermore, we define elasticity of demand with respect to quality and procedure volume as:

7 $$ {E}_{jt}^{K_{j,t-2}}={\sum}_{i\in {S}_{jt}}\frac{{\partial P}_{ijt}}{{\partial K}_{\mathrm{j},t-2}}\frac{K_{j,t-2}}{\sum_{i\in {S}_{jt}}{P}_{ijt}}={\sum}_{i\in {S}_{jt}}{\beta}_k{P}_{ijt}\left(1-{P}_{ijt}\right)\frac{K_{j,t-2}}{\sum_{i\in {S}_{jt}}{\beta}_k{P}_{ijt}} $$

for *K* = {*Q*, *PV*} and *β*_*k*_ = {*β*_*q*_, *β*_*pv*_}. We report the average of (7) weighted by each hospital’s relative importance, i.e. their expected demand ${\sum}_{i\in {S}_{jt}}{P}_{ijt}$.

Lastly, we want to evaluate the effect of quality competition on hospital demand. Thus, we calculate cross-elasticities with respect to competing hospitals *j*′ quality ${Q}_{j^{\prime }t-2}$, where *j* ′  ≠ *j*.

8 $$ {E}_{jt}^{Q_{j^{\prime }}}={\sum}_{i\in {S}_{jt}\cap {S}_{j^{\prime }t}}\frac{{\partial P}_{ijt}}{{\partial Q}_{j^{\prime },t-2}}\frac{Q_{j^{\prime },t-2}}{\sum_{i\in {S}_{jt}}{P}_{ijt}}={\sum}_{i\in {S}_{jt}\cap {S}_{j^{\prime }t}}{\beta}_q{P}_{ijt}\left(1-{P}_{i{j}^{\prime }t}\right)\frac{Q_{j^{\prime },t-2}}{\sum_{i\in {S}_{jt}}{\beta}_q{P}_{ijt}} $$

If hospital *j*_1_ does not have patients overlapping with hospital *j*_2_, then its cross-elasticity is 0 by default. Demand responsiveness and elasticities are calculated separately for each procedure.

## Results

### Descriptive statistics

Our two samples consist of 66,645 patients treated in 862 different hospitals in 2017 and 2018 for colorectal resection, resulting in 1617 hospital observations, and 82,041 patients treated in 844 different hospitals resulting in 1591 hospital observations for knee replacement, respectively. Descriptive statistics are shown in Table 1.

**Table 1:** Descriptive statistics

Variables	Obs.	Mean	Median (IQR)	Std. Deviation
**Colorectal resection**				
*Patient-level (2017, 2018)*				
Travel time [min]	66,645	19.4	15.4 (8.6–24.9)	16.3
Travel time past closest hospital [min]	66,645	7.0	0.1 (0–8.3)	13.4
Number of hospitals in 10 min radius	66,645	1	0 (0–1)	1
Number of hospitals in 30 min radius	66,645	8	4 (2–10)	9
Number of hospitals in 60 min radius	66,645	34	24 (15–39)	32
Age	66,645	67	69 (58–79)	16
Charlson comorbidity index	66,645	3.55	2 (0–6)	5.05
Median income [€]	66,645	3074	3127 (2873-3323)	420
*Hospital-level (2015, 2016)*				
Patient recommendation	1617	1.97	1.96 (1.77–2.16)	0.29
90-day mortality ratio	1617	1.05	0.88 (0.35–1.49)	1.00
Medical interdisciplinarity	1617	0.95	1 (1–1)	0.19
Medical therapy offerings	1617	0.57	0.5 (0.5–1)	0.37
Procedure volume	1617	131	108 (68–175)	92
*Hospital-level (2017, 2018)*				
Cancer treatment center	1617	0.31	0 (0–1)	–
Professional recommendation (award)	1617	0.10	0 (0–0)	–
**Knee replacement**				
*Patient-level (2017, 2018)*				
Travel time [min]	82,014	24.5	20.4 (11.9–32.5)	18.1
Travel time past closest hospital [min]	82,014	11.1	4.9 (0–17)	15.7
Number of hospitals in 10 min radius	82,014	1	0 (0–1)	1
Number of hospitals in 30 min radius	82,014	7	4 (2–8)	7
Number of hospitals in 60 min radius	82,014	29	22 (14–36)	24
Age	82,014	69	70 (62–77)	10
Charlson comorbidity index	82,014	0.70	0 (0–1)	1.16
Median income [€]	82,014	3064	3125 (2872-3317)	419
*Hospital-level (2015, 2016)*				
Patient recommendation	1591	1.89	1.90 (1.69–2.1)	0.31
1-year revision rate	1591	1.04	0.82 (0–1.55)	1.13
Medical interdisciplinarity	1591	0.74	0.8 (0.5–1)	0.24
Medical therapy offerings	1591	0.49	0.7 (0.3–0.7)	0.26
Procedure volume	1591	160	114 (72–197)	141
*Hospital-level (2017, 2018)*				
Endoprosthetic care center	1591	0.50	0 (0–1)	–
Professional recommendation (award)	1591	0.11	0 (0–0)	–

Obs = Observations; IQR = Interquartile range. *Notes*: Hospital features are unweighted. Scores for patient recommendation are from 1 (best score) to 6 (worst score). Professional recommendation is expressed as either being decorated with a top hospital award = 1 or not = 0. Hospital observations are counted per hospital and year

Hospitals treating colorectal resection patients perform, on average, around 131 procedures per year, whereas 25% of hospitals treat more than 68 patients. Around 30% of hospitals are certified, and around 10% of hospitals are decorated with a top hospital award. Average annual procedure volume for knee replacement hospitals is 160 and 50% of hospitals treat at least 114 patients and are certified. Roughly 11% of hospitals are decorated with a top hospital award. Both colorectal resection and knee replacement patients are on average in their late sixties (67 and 69 respectively) but colorectal resection patients are characterized by a significant higher comorbidity index than knee replacement patients (3.54 vs. 0.70).

**Fig. 2:**
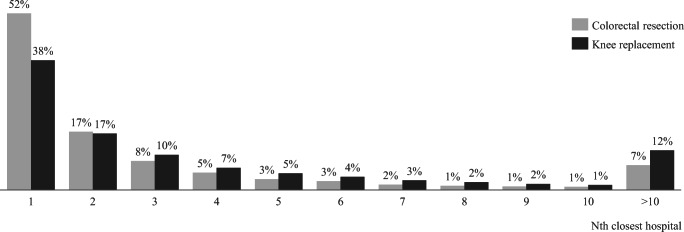
Share of patients that chose their Nth closest hospital

Regarding travel time, the average colorectal resection (knee replacement) patient travels around 19 (25) minutes to receive treatment and around 7 (11) minutes past the closest hospital. More than 50% of colorectal resection patients choose their closest hospital for treatment (see Fig. 2). Interestingly, the travel time associated with the patient’s proximity choice is individual and systematically different between rural and urban patients. For instance, some rural patients that choose their closest hospital must travel 70 min whereas some urban patients that choose their 10th closest hospital travel less than 10 min (see Figs. 8 and 9 in the supplementary material).

Furthermore, with respect to colorectal resection, patient recommendation correlates slightly with 90-day mortality (ρ = 0.07) and medical therapy offerings (ρ = −0.11) as well as number of beds (ρ = 0.15). 90-day mortality correlates slightly with procedure volume (ρ = −0.08) and certification (ρ = −0.08). While these correlations are rather negligible, we observe high correlations between number of beds and procedure volume (ρ = 0.70), certification (ρ = 0.47), professional recommendation (ρ = 0.55) and university hospital (ρ = 0.63). To rule out biased estimators in our econometric model we also estimated a model without the hospital variable number of beds but discovered no difference in marginal utilities.

Regarding knee replacement, we discovered moderate correlations between patient recommendation and procedure volume (ρ = −0.42), professional recommendation (ρ = −0.23), and inversely for number of beds (ρ = 0.24). 1-year revision only correlates slightly with procedure volume (ρ = −0.09) and patient recommendation (ρ = 0.08). Other moderately high correlations are observed between procedure volume and professional recommendation (ρ = 0.54), and number of beds and university hospital (ρ = 0.62). Surprisingly, procedure volume and number of beds are inversely correlated (ρ = −0.09), indicating that knee replacement patients are often treated in specialized centers that are smaller in size [1]. Figs. 10 and 11 in the supplementary material show the full correlation matrices.

### Regression results

Before being able to interpret estimated marginal utilities for our basic MNL model, we test for the independence of irrelevant alternatives (IIA) assumption to hold. For both models the Hausman-McFadden Test does not reject the IIA assumption. We can therefore proceed with the interpretation of the results of our basic model. Marginal utilities and WTT are reported in Tables 2 and 3.

**Table 2:** Estimated marginal utilities for colorectal resection

Variable	Estimate	Standard Error	
*Main effects*			
Travel time	−0.203	0.002***	
Travel time^2^	0.001	0.000***	
Travel time^3^	0.000	0.000***	
Patient recommendation	−0.226	0.022***	
90-day mortality ratio	−0.040	0.006***	
Medical interdisciplinarity	0.168	0.032***	
Medical therapy offerings	0.062	0.016***	
Procedure volume	0.003	0.000***	
Certification	0.316	0.013***	
Professional recommendation (award)	−0.035	0.018	
Hospital type: non-profit vs. private	−0.127	0.019***	
Hospital type: public vs. private	0.171	0.017***	
*Interaction with travel time*			
x Age	−0.001	0.000***	
x Comorbidity index	0.000	0.000	
x Number of hospitals in 30 min radius	−0.004	0.000***	
x Median income	0.000	0.000*	
*Interaction with patient recommendation*			
x Age	0.005	0.001***	
x Comorbidity index	0.028	0.004***	
x Number of hospitals in 30 min radius	0.011	0.002***	
x Median income	0.000	0.000***	
*Interaction with 90-day mortality*			
x Age	0.000	0.000	
x Comorbidity index	−0.001	0.001	
x Number of hospitals in 30 min radius	0.001	0.001	
x Median income	0.000	0.000***	
*Interaction with medical interdisciplinarity*			
x Age	−0.003	0.002	
x Comorbidity index	0.014	0.007*	
x Number of hospitals in 30 min radius	−0.016	0.003***	
x Median income	0.000	0.000***	
*Interaction with medical therapy offering*			
x Age	0.001	0.001	
x Comorbidity index	−0.002	0.003	
x Number of hospitals in 30 min radius	−0.005	0.002**	
x Median income	0.000	0.000	
*Interaction with procedure volume*			
x Age	0.000	0.000	
x Comorbidity index	0.000	0.000***	
x Number of hospitals in 30 min radius	0.000	0.000***	
x Median income	0.000	0.000***	
*Interaction with certification*			
x Age	−0.001	0.001	
x Comorbidity index	0.009	0.002***	
x Number of hospitals in 30 min radius	0.003	0.001*	
x Median income	0.000	0.000***	
*Interaction with professional recommendation (award)*			
x Age	−0.009	0.001***	
x Comorbidity index	0.017	0.003***	
x Number of hospitals in 30 min radius	−0.006	0.002**	
x Median income	0.000	0.000***	
			*As % of average travel time*
WTT (recommendation)	−0.384		*2.0%*
WTT (90-day mortality)	−0.232		*1.2%*
WTT (medical interdisciplinarity)	0.180		*0.9%*
WTT (medical therapy offering)	0.129		*0.7%*
WTT (procedure volume)	1.800		*9.3%*
WTT (certification)	1.820		*9.4%*
Number of patients	66,645		
Number of hospitals	862		
Prob > chi^2^	0.000		
Pseudo R^2^	0.587		

*Notes*: Multinomial logit model for colorectal resection patients treated in 2017 and 2018. Coefficients represent marginal utilities. Coefficients of the covariates number of beds, university hospital status and their interactions with patient features are not reported here but available on request along with travel time^2^ and travel time^3^ and hospital type interactions with patient features

*** *p* < 0.001; ** *p* < 0.01; * *p* < 0.05

Regarding colorectal resection, the reference patient favors shorter travel times and prefers hospitals with a better patient recommendation, lower 90-day mortality, a higher degree of medical interdisciplinarity and a broad offer of medical special therapies. Further, patient utility is positively affected by specialization, i.e. both by higher procedure volume and by certification. The effect of professional recommendation is not significant. The reference patient prefers public over private over non-profit hospitals. Further, the older and the more options the patient has in proximity, the greater is the disutility of travelling.

The estimated WTT from changes in quality or specialization differ between indicators. Specialization in form of higher procedure volume (+ 9.3% of average travel time) and certification (+ 9.4%) have the highest impact. The effects of a one SD reduction of patient recommendation and 90-day mortality are significant but rather small (+2.0 and + 1.2%); the WTT of the structural quality indicators are very small (+0.7% and + 0.9%) and will therefore be neglected in further analyses.

Similar effects can be observed for knee replacement patients. The reference patient favors shorter travel times and prefers a higher patient recommendation score and a lower 1-year revision rate but does not have a significant preference towards full medical interdisciplinarity or medical therapy offerings. In contrast, higher specialization, expressed by higher procedure volume and certification, increases the reference patient’s utility. Moreover, the knee replacement patient cares about professional recommendation and favors private over public over non-profit hospitals. Again, with more choices in proximity and increased age and comorbidity, traveling for treatment creates more disutility.

Specialization has a major impact on knee replacement patients’ hospital choice. The WTT for a one SD increase in procedure volume and for being certified are ~3.1 and 1.3 min or 12.5% and 5.3% of average patient travel time. Contrary to colorectal resection, the impact of improvement in recommendation measures for knee replacement patients is rather high. WTT for a one SD improvement in patient recommendation and for being awarded a top hospital through professional recommendation are 1.4 and 0.6 min or 5.6% and 2.3% of average patient travel time. The 1-year revision rate is significant but triggers a relatively small additional WTT (~0.2 min).

Additionally, we estimate time-invariant hospital fixed effect MNL models for colorectal resection and knee replacement. Results are shown in Table A3 in the supplementary material. Results show marginal utility effects of quality changes in the observed time span. For colorectal resection, we can see that only travel time and patient recommendation are significantly affecting patients’ hospital choice. All other variables are insignificant. With regard to knee replacement, this insignificance is quite drastic; only travel time has a significant negative effect on patients’ utility and we therefore cannot calculate any meaningful WTT.

The variables’ insignificance is most likely due to limited within-hospital variation over time. Further, the fixed effects probably absorb large parts of the time-invariant absolute quality. For example, structural quality indicators as well as procedure volume usually do not fluctuate significantly over a two-year time horizon. Nevertheless, the effect of patient recommendation score of the time-invariant hospital fixed effect model in the patient decision-making process is very similar to the basic model (WTT of 1.9 min or 2.0% of average travel time).

As sensitivity analyses, we also estimate a model excluding procedure volume from our MNL model (see Table A4 in the supplementary material). As expected, effects of all quality metrics are inflated due to their positive correlation with procedure volume. Regarding colorectal resections, we observe that the effect is mostly absorbed by certification (WTT of 9.3% vs. 14.0%) and patient recommendation (WTT of 2.0% vs. 3.2%). With regard to knee replacement, the effect of procedure volume on patients’ hospital choice is especially absorbed by professional recommendation (WTT of 2.3% vs. 24.6%), in line with its high positive correlation (*ρ* = 0.54). Moreover, the effect of patient recommendation (WTT of 5.6% vs. 11.2%) and of 1-year revision rate (WTT of 0.7% vs. 2.5%) increases considerably when excluding procedure volume.

### Demand effects

Table 4 shows the demand effects for quality changes for both procedures. We concentrate on the quality indicators whose estimates are significant and whose effect on demand is noteworthy. We estimate demand changes triggered by changes in quality and express them in terms of the average number of AOK patients treated per hospital, extrapolate the demand change to the overall German population, and calculate the indicators’ demand elasticity where appropriate.

**Table 3:** Estimated marginal utilities for knee replacement

Variable	Estimate	Standard Error	
*Main effects*			
Travel time	−0.135	0.001***	
Travel time^2^	0.000	0.000***	
Travel time^3^	0.000	0.000***	
Patient recommendation	−0.539	0.017***	
1-year revision rate	−0.019	0.005***	
Medical interdisciplinarity	−0.008	0.019	
Medical therapy offerings	−0.040	0.017*	
Procedure volume	0.003	0.000***	
Certification	0.159	0.009***	
Professional recommendation (award)	0.070	0.014***	
Hospital type: non-profit vs. private	−0.218	0.012***	
Hospital type: public vs. private	−0.152	0.012***	
*Interaction with travel time*			
x Age	−0.001	0.000***	
x Comorbidity index	−0.004	0.001***	
x Number of hospitals in 30 min radius	−0.003	0.000***	
x Median income	0.000	0.000**	
*Interaction with recommendation*			
x Age	0.009	0.002***	
x Comorbidity index	0.044	0.015**	
x Number of hospitals in 30 min radius	−0.001	0.002	
x Median income	0.000	0.000***	
*Interaction with 1-year revision rate*			
x Age	−0.001	0.000	
x Comorbidity index	−0.004	0.004	
x Number of hospitals in 30 min radius	−0.001	0.000	
x Median income	0.000	0.000**	
*Interaction with medical interdisciplinarity*			
x Age	0.001	0.002	
x Comorbidity index	0.015	0.017	
x Number of hospitals in 30 min radius	0.027	0.003***	
x Median income	0.000	0.000***	
*Interaction with medical therapy offering*			
x Age	0.004	0.002*	
x Comorbidity index	0.124	0.016***	
x Number of hospitals in 30 min radius	−0.029	0.002***	
x Median income	0.000	0.000***	
*Interaction with procedure volume*			
x Age	0.000	0.000***	
x Comorbidity index	0.000	0.000***	
x Number of hospitals in 30 min radius	0.000	0.000**	
x Median income	0.000	0.000***	
*Interaction with certification*			
x Age	0.000	0.001	
x Comorbidity index	0.042	0.008***	
x Number of hospitals in 30 min radius	−0.008	0.001***	
x Median income	0.000	0.000	
*Interaction with professional recommendation (award)*			
x Age	−0.009	0.001***	
x Comorbidity index	0.050	0.012***	
x Number of hospitals in 30 min radius	0.010	0.002***	
x Median income	0.000	0.000	
			*As % of average travel time*
WTT (patient recommendation)	1.370		*5.6%*
WTT (1-year revision)	0.171		*0.7%*
WTT (procedure volume)	3.066		*12.5%*
WTT (certification)	1.297		*5.3%*
WTT (professional recommendation)	0.571		*2.3%*
Number of patients	82,014		
Number of hospitals	844		
Prob>chi^2^	0.000		
Pseudo R^2^	0.478		

*Notes*: Multinomial logit model for colorectal resection patients treated in 2017 and 2018. Coefficients represent marginal utilities. Coefficients of the covariates number of beds, university hospital status and their interactions with patient features are not reported here but available on request along with travel time^2^ and travel time^3^ and hospital type interactions with patient features

*** *p* < 0.001; ** *p* < 0.01; * *p* < 0.05

**Table 4:** Demand responsiveness and elasticity of demand

Procedures	Indicator	Observed		Marginal utility	Effect of change in quality or specialization					Elasticity
		Mean	SD		WTT	% WTT of mean TT	Demand change	% demand change	Demand change†	
Colorectal resection	Patient recommendation	1.97	0.29	0.242	0.38	2.0%	1.69	4.1%	5.0	0.27
	90-day mortality ratio	1.05	1.00	−0.023	0.23	1.2%	1.02	2.5%	3.0	0.02
	Procedure volume	131	92	0.003	1.80	9.3%	7.92	19.2%	23.5	0.37
	Certification	0.31	–	0.295	1.82	9.4%	8.01	19.4%	23.7	–
Knee replacement	Patient recommendation	1.89	0.31	0.539	1.37	5.6%	6.36	12.3%	18.4	0.70
	1-year revision rate	1.04	1.13	−0.019	0.17	0.7%	0.80	1.5%	2.3	0.01
	Procedure volume	160	141	0.003	3.07	12.5%	14.23	27.6%	41.2	0.45
	Certification	0.50	–	0.159	1.30	5.3%	6.02	11.7%	17.4	–
	Professional recommendation	0.11	–	0.070	0.57	2.3%	2.65	5.1%	7.7	–

SD = Standard Deviation; TT = Travel Time. *Notes*: Marginal utilities, WTT and elasticity of the inversely rated indicator patient recommendation and clinical quality indicators is shown as positive number to support more intuitive interpretation. Demand change† denotes the extrapolated demand change (demand change divided by ratio of patient sample and total procedure volume 2017 & 2018).

Changes in the degree of specialization of the colorectal resection performing hospitals in form of procedure volume and certification show the largest demand effects. By increasing the procedure volume by one SD, hospitals can on average create 19% additional change in demand. This demand increase should be taken with care, as the SD of procedure volume is very large. Being certified increases the demand of colorectal resection hospitals on average by around 19%. Extrapolating the demand change would lead on average to 24 additional patients from competing hospitals after receiving certification. An improvement of the patient recommendation score by one SD leads to a moderate demand increase of around 4%. In contrast, a one SD decrease of the 90-day mortality ratio barely affects hospital demand. Demand elasticities show the effect of relative improvements of the quality indicators on demand, qualifying the size of each SD. Regarding colorectal resection, results show that a 1% improvement in patient recommendation would lead to a 0.27% increase in demand. Similarly, increasing procedure volume by 1% would increase demand by 0.37%. The demand elasticity of 90-day mortality rate is close to zero and thus almost irrelevant.

Results for knee replacement show some similarities, but also some differences can be observed. Besides a large effect by increasing specialization, 27% demand increase through a one SD increase in procedure volume and an 11% demand increase by certification, recommendation by patients (12% demand change) and by physicians (5% demand change) play an important role. Again, procedure volume constitutes a very large SD, which should be considered in its interpretation. Patient recommendations play an important role for knee replacement which becomes even more transparent when looking at demand elasticities: a 1% improvement in patient recommendation leads to a 0.70% increase in demand, whereas a 1% increase in procedure volume raises demand by 0.45%.

**Fig. 3:**
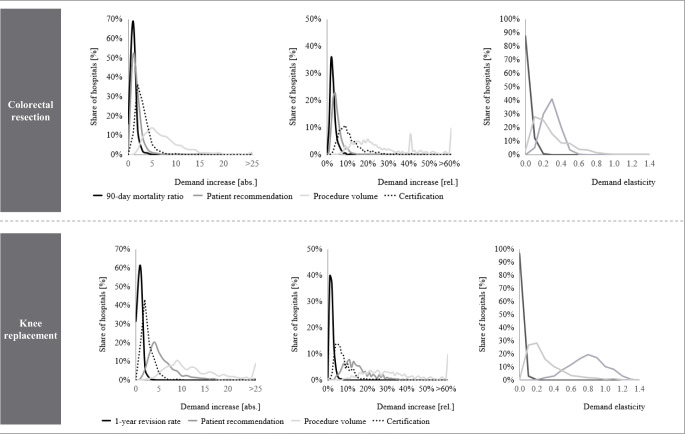
Absolute and relative demand gain and demand elasticities for selected indicators Notes: For the indicator certification, only hospitals without an existing certification were considered. Absolute and relative demand increase are calculated only with the investigated patient sample and are not extrapolated. Increases are expressed for a one standard deviation improvement in quality. For certifications, increases signal a possible demand gain from receiving a certification

Additionally, Fig. 3 details average demand effects and illustrates the distribution of absolute and relative demand increases and elasticities for all hospitals. Whereas the cross-hospital variance in 90-day mortality, patient recommendation for colorectal resections, 1-year revision rate and endoprosthetic certification is limited, we observe larger variances for the demand effects of procedure volumes, certification for colorectal resection and patient recommendation for knee replacements.

**Fig. 4:**
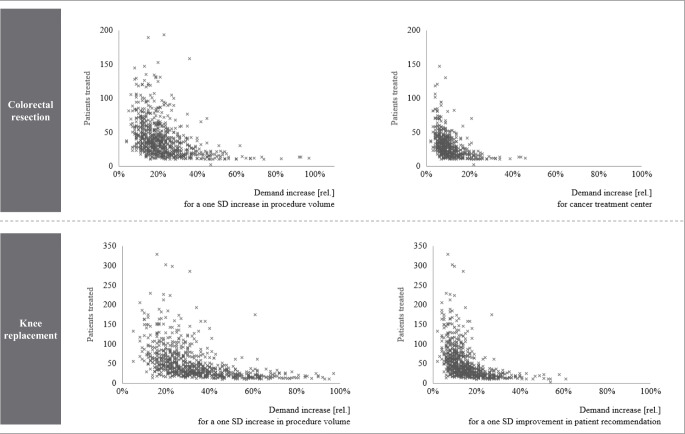
Relative demand increase per hospital by hospital patient volume Notes: For the indicator certification, only hospitals without an existing certification were considered. Demand increases are calculated only with the investigated patient sample and are not extrapolated; Patients treated is defined as the average number of AOK patients treated by a hospital in 2017 and 2018

In Fig. 4 we investigate these larger cross-hospital variances and plotted relative demand effects against the number of AOK patients treated in each hospital. We find that the impact of changes with respect to procedure volume, certification (colorectal resection) and patient recommendation score (knee replacement) is especially high for hospitals that treat fewer patients.

Moreover, when mapping results of relative demand changes into the accessibility – comprehensibility matrix we can see a general trend. Quality indicators such as procedure volume, certification and patient recommendation that score high in comprehensibility and accessibility show the largest demand effects. The clinical quality indicators 90-day mortality ratio and 1-year revision rate having negligible demand effects are hard to interpret for medical laymen and are partly not even accessible for patients and physicians (see Fig. 12 in the supplementary material).

### Competition effects

**Fig. 5:**
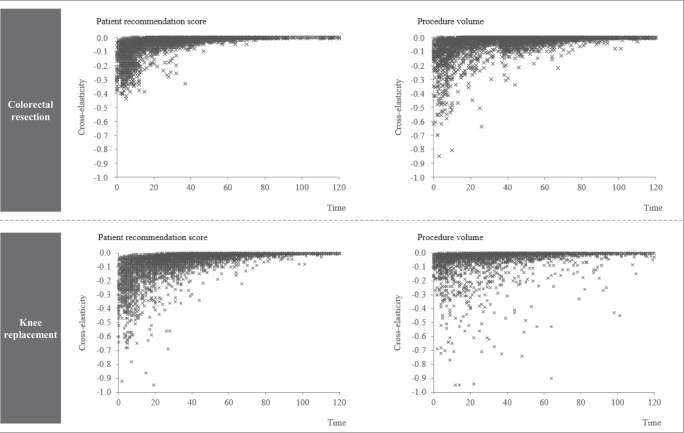
Cross-elasticity with respect to improvements of quality of competing hospitals vs. travel time between hospitals Notes*:* Due to clarity, time is rounded to minutes and several similar data points are shown as a single marker. Six data points with cross-elasticities smaller than −1 are not displayed for the indicator procedure volume for knee replacement

Lastly, in a market with quality competition, there are also reciprocal effects between hospitals, i.e. when one hospital improves the quality it will most likely affect the demand of nearby hospitals negatively (see also [21]). In Fig. 5 we can observe that cross-elasticities are stronger the closer hospitals are located to each other. The average cross-elasticity for hospitals within 20-min distance for patient recommendation and procedure volume are −0.060 and − 0.070 for colorectal resection, and − 0.107 and − 0.063 for knee replacement. These cross-elasticities decrease to −0.005 and − 0.006 (colorectal resection) and to −0.016 and − 0.010 (knee replacement) for hospitals that are located in between 20 to 60 min driving time from each other.

## Discussion

Quality differences between hospitals affect patients’ hospital choice. The magnitude and significance of these effects differ between indicators and procedures. In general, we see that higher patient recommendation scores as well as a higher degree of specialization, namely procedure volume and certification, have a significant positive impact on patients’ hospital choice. Structural quality indicators and clinical quality indicators are significant for patients’ hospital choice yet their effect on WTT and hospital demand is negligible.

Results also reveal that patients value different quality metrics for different procedures. Regarding colorectal resection, specialization has the strongest effect on patients’ hospital choice whereas knee replacement hospitals can, besides specializing, achieve significant demand effects by improving patient and physician recommendations. Further, results show that colorectal cancer treatment center certifications are being awarded more restrictively than those for endoprosthetic care centers and therefore seem to have a better signaling effect for patients as its impact on patients’ hospital choice is considerably stronger. The impact of colorectal cancer treatment center certifications is even more remarkable considering that only around 61% of colorectal resections are performed on patients diagnosed with cancer. When we reestimate our model only with colorectal resection patients diagnosed with cancer, we observe a considerably larger WTT of 12.6% for being certified (see Table A5 in the supplementary material).

Moreover, and as expected, quality metrics with higher accessibility and comprehensibility tend to show larger impact on patients’ hospital choice with respect to WTT and hospital demand. For example, patient recommendation scores are transparently displayed on public online platforms and are easy to interpret, even for medical laymen. Clinical outcome quality indicators are not published for colorectal resection and generally very difficult to comprehend [29]. Professional recommendation, at least in form of hospital awards, can be considered as easy to interpret for patients as patient recommendation, but surprisingly, its effect is either small (knee replacement) or not significant (colorectal resection). Rather high correlations between professional recommendation and procedure volume, certification and university hospital status could indicate that this quality metric does not add new and incremental information for the patient [7], at least partially explaining our results. This is underlined by comparing our main model to the model excluding procedure volume; especially for knee replacement, professional recommendation seems to be mostly determined by procedure volume. It can be concluded that patients use information on quality and specialization rationally but only if information is presented transparently and comprehensibly. Still, it is essential that hospitals do not solely concentrate on the improvement of service quality at the cost of clinical quality [36].

Further, we can see that demand effects vary across hospitals depending on selected prior characteristics. Hospitals with fewer AOK patients tend to have larger relative demand increases with respect to quality improvements compared to hospitals already treating a relatively high number of patients. Moreover, quality competition can be observed for selected indicators. Quality improvement or higher specialization of competing hospitals nearby affects a hospital’s demand negatively. Comparison of the two procedures reveals that competition for colorectal resection is rather local, whereas hospitals for knee replacement also face regional competition.

### Findings from other studies

Various other studies have investigated the effect of quality metrics on patients’ hospital choice. Most studies differ either in geography [21], investigated procedure [19] or both [9]. A direct and detailed comparison is therefore difficult, as spatial differences and different procedures affect estimated results. For example, the UK, the Netherlands, and Germany differ in their degree of hospital centralization, population density, and competition [37]. Further, our results show that the degree to which quality information impacts patients’ hospital choice is conditional on their procedure. Nevertheless, we consider our estimations to be in line with comparable studies.

Gutacker et al. [21] employ a conditional logit model to estimate determinants for patients’ hospital choice for hip replacement in the UK. On the one hand, the authors find that the clinical outcome indicators 1-year revision rate and 28-day mortality rate are not significant for patients’ hospital choice. On the other hand, 28-day readmission rate and a change in patient-reported outcome measures (PROM) scores impact both the patients’ WTT and hospital demand. Patients are willing to travel an additional 0.9 km or 6% of the average distance for a one SD improvement in the PROM change. Regarding emergency readmission, patients are willing to travel 0.6 km or 4% longer for a one SD improvement of the 28-day emergency readmission rate.

Avdic et al. [19] conclude that for maternal care services in Germany for a one SD improvement of clinical process and outcome quality indicators (decision-to-delivery interval, availability of pediatrician, perineal tear) mothers are willing to travel between 0.2 and 0.6 km longer (2 and 6% of average travel time). WTT for a one SD improvement in service quality are as high as 1.6 km or 15% for medical and nursing services and 0.5 km or 5% for patient recommendation. Varkevisser et al. [9] examine the effect of professional recommendation and clinical outcome quality on patients’ hospital choice for angioplasty in the Netherlands. Their results show that patients are willing to travel 2 min or 9% of average travel time longer for a 1 percentage point lower readmission rate. Considering the different procedures and geographies, we find out results for knee replacement and colorectal resection to be in line with the aforementioned studies.

### Limitations

Patient data are collected by patients’ health insurers and a comprehensive data set comprising all patients of a certain procedure as well as patient and hospital location references is not available. Thus, like other German studies on patients’ hospital choice (e.g. [19, 20]) our sample only covers a fraction of all German patients. Nevertheless, the use of AOK patient-level data for 2017 and 2018 allows us to cover approximately 35% of German colorectal resection and knee replacement patients, representing the largest German data set available. As it can be assumed that patient characteristics do not vary considerably between sickness funds, we are confident that our estimates are generalizable.

Furthermore, in our model we do not account for unobserved patient characteristics like individual preferences or differences in individual capability to access and understand quality information. As shown by Gutacker et al. [21], a random coefficient MNL model controls for this patient heterogeneity and relaxes the assumption for the independence of irrelevant alternatives (IIA). As computation time for such models is excessive for large data sets and as our Hausman-McFadden test did not reject the IIA assumption, we continued with the regular MNL model. Besides, Gutacker et al. [21] report that results of both MNL models showed similar effects. Further, in the presence of unobserved effects on patients’ hospital alternatives, a nested logit model is an interesting alternative to use, as it accounts for similarities between alternatives via partial correlation of the error terms, and thus relaxes the IIA assumption [38]. Given an efficient development of nests for patients’ alternatives, this model promises robust results, when the IIA assumption is in doubt. Due to no apparent grouping of hospitals and our results for the Hausman-McFadden test we resumed our analyses with the regular MNL model.

Moreover, results of the time-invariant hospital fixed effect model show insignificant effects for most indicators. This does not mean that quality information has no influence on patient choice, but rather reveals that quality metrics’ within-hospital variance over time is rather small. These results are in line with Gutacker et al. [21] and Avdic et al. [19]. To isolate the impact of quality signaling efficiently, a time-invariant hospital fixed effect model should be set up with panel data over a period of 5 to 10 years.

Additionally, despite the inclusion of professional recommendation as a proxy for physicians’ referral patterns, there is no reliable metric to measure referring physicians’ impact on patients’ hospital choice properly. Nevertheless, (clinical) quality should influence physicians’ referrals at least to some degree [29].

## Conclusion

In health care markets with free choice and hospitals acting as economic entities, hospital management is incentivized to distribute budgets according to the most effective patient attraction. Our study shows that patients are considerably attracted to more specialized hospitals with higher procedure volumes, certification and high patient recommendation scores (colorectal resection) and specialized hospitals, which are highly recommended by patients and physicians (knee replacement). At the same time, patients only respond weakly to clinical outcome (both procedures) and structural quality indicators (both procedures). Further, the degree and significance of impact can be explained by the accessibility and comprehensibility of these indicators.

Hospital management needs to be informed of the economic benefits from quality competition. Our results show that hospitals should pursue a differentiated strategic approach to maximize demand based on the nature of their procedures. For instance, colorectal resection hospitals are economically incentivized to specialize further (increased procedure volume, certification) and to improve their service quality (patient recommendation score). Knee replacement hospitals should focus on service quality by improving recommendation scores and, moreover, should specialize further. Moreover, specialization might also improve clinical outcome quality, e.g. due to more experienced surgeons and post-operative care teams. While some quality improvements have procedure-specific demand effects, others like service quality and hospital reputation might affect all procedures of a hospital.

Most importantly, policy makers should utilize the insights generated by this study to shape patients’ hospital choice by incentivizing competition on all types of quality. Consequently, transparency and understanding of outcomes need to be improved. For instance, sickness funds should cooperate with health ministries to enhance existing online quality platforms, such as *weisseliste.de* or the *AOK Krankenhaus Navigator* to build a central comprehensive platform for hospital quality information. This platform should be extensively promoted in cooperation with referring physicians. In addition, relevant outcome quality indicators need to be defined [26]. PROMs show promising potential in measuring treatment outcomes that is relevant for patients both for orthopedic and oncologic procedures [39, 40]. All those quality information needs to be presented understandably and in detail. Lastly, quality changes over time need to be visible in order to enable patients to adjust their hospital choice accordingly.

## Supplementary Information

ESM 1 (DOCX 478 kb)

## Abbreviations

MNL: Multinomial Logit
IIA: Independence of Irrelevant Alternatives
WTT: Willingness To Travel
FE: Fixed Effect
SD: Standard Deviation
PROM: Patient-Reported Outcome Measure

## Footnotes

1 We consider the certification for colorectal cancer treatment center and hospital awards for colorectal cancer treatment to be the most suitable indicators for the procedure colorectal resections as 61% of patients receiving colorectal resections are cancer patients. Moreover, colorectal cancer treatment center is listed as relevant certification for the procedure colorectal resections on central online hospital search platforms, e.g. https://weisse-liste.krankenhaus.aok.de/.

2 For the full list of centers see https://oncomap.de/centers

3 For the full list of centers see https://endocert.de/

4 Recommendation is addressed by question no. 14: “Would you recommend this hospital to your best friend?”

5 Relevant medical interdisciplinarity for colorectal resection comprises medical specialty such as general surgery and visceral surgery. Relevant medical interdisciplinarity for knee replacement comprises medical specialty such as general surgery, orthopedic and trauma surgery, special orthopedic surgery and special trauma surgery.

6 Medical therapy offerings for colorectal resection are dieticians and stoma therapy. Medical therapy offerings for knee replacement are physiotherapist, masseuse, and orthopedic mechanic.

7 Data are collected in year 0, processed and checked in year 1, and published in year 2

8 As example see https://weisse-liste.krankenhaus.aok.de/

9 An evaluation based on three clinical quality indicators is available, for detailed information on 1-year revision several clicks are necessary, however.

10 In other words: “How much longer is the reference patient willing to travel for an increase in quality?”
