# Supplementary material for: The impact of quality on hospital choice. Which information affects patients’ behavior for colorectal resection or knee replacement?
Source: Health Care Manag Sci. 2021 Jan 27;24(1):185–202. doi: 10.1007/s10729-020-09540-2 (PMC8184721; doi:10.1007/s10729-020-09540-2)
Supplement: Supplementary file 1 — (DOCX 478 kb) [file 10729_2020_9540_MOESM1_ESM.docx]

# Supplementary Material

Table A1: Definition of the procedure colorectal resection

| Source | Definition of procedure  (Colon resection) | Definition of procedure  (Rectum resection) |
| --- | --- | --- |
| German Society for General and Visceral Surgery | 5-455, 5-456 | 5-484, 5-485, 5-486.3, .4 |

*Notes*: Procedure codes are part of the German procedure catalogue (*Operationen- und Prozedurenschluessel*). Procedure code 5-458 for Colon Resection still is listed as part of the medical society’s definition for colon resection yet this procedure code has not been valid since 2012 (see https://www.dimdi.de/static/de/klassifikationen/ops/kode-suche/opshtml2012/).

Sources: DGAV [1], WidO [2]

Table A2: Definition of total knee replacement

| Source | Inclusion criteria procedure |
| --- | --- |
| Federal Joint Committee | 5-822.9, 5-822.g, 5-822.h,  5-822.j, 5-822.k |

Source: Gemeinsamer Bundesausschuss (G-BA) [3]

Table A3: Estimated marginal utilities – time-invariant hospital fixed effects

| **Colorectal resection** | Estimate | Standard Error |  |
| --- | --- | --- | --- |
| *Main effects* |  |  |  |
| Travel time | -0.218 | 0.002*** |  |
| Patient recommendation | -0.251 | 0.092** |  |
| 90-day mortality ratio | -0.005 | 0.011 |  |
| Medical interdisciplinarity | -0.393 | 0.314 |  |
| Medical therapy offerings | -0.185 | 0.396 |  |
| Procedure volume | -0.001 | 0.000 |  |
| Certification | 0.741 | 0.390 |  |
| Professional recommendation (award) | 0.001 | 0.177 |  |
|  |  |  | *As % of average travel time* |
| WTT (patient recommendation) | 1.876 |  | *2.0%* |
| Number of patients | 64,027 |  |  |
| Number of hospitals | 755 |  |  |
| Prob>chi^2^ | 0.000 |  |  |
| Pseudo R^2^ | 0.619 |  |  |
| **Knee replacement** |  |  |  |
| *Main effects* |  |  |  |
| Travel time | -0.144 | 0.002*** |  |
| Patient recommendation | -0.066 | 0.070 |  |
| 1-year revision rate | 0.000 | 0.008 |  |
| Medical interdisciplinarity | -0.426 | 0.259 |  |
| Medical therapy offerings | -0.475 | 0.337 |  |
| Procedure volume | 0.000 | 0.000 |  |
| Certification | -0.083 | 0.052 |  |
| Professional recommendation (award) | 0.217 | 0.177 |  |
| Number of patients | 78,786 |  |  |
| Number of hospitals | 747 |  |  |
| Prob>chi^2^ | 0.000 |  |  |
| Pseudo R^2^ | 0.522 |  |  |

Notes: Conditional logit model of hospital choice for colorectal resection / knee replacement patients treated in 2017 and 2018. Coefficients represent marginal utilities. Coefficients of the covariates number of beds, hospital type, university hospital status and their interactions with patient features are not reported here but available on request along with each variable’s interactions with patient features. The hospital sample was restricted to only those hospitals that passed the data cleaning steps described in Figure 6 for both years.

*** p < 0.001; ** p < 0.01; * p < 0.05

Table A4: Estimated marginal utilities – MNL without procedure volume

| **Colorectal resection** | Estimate | Standard Error |  |
| --- | --- | --- | --- |
| *Main effects* |  |  |  |
| Travel time | -0.203 | 0.002*** |  |
| Patient recommendation | -0.370 | 0.022*** |  |
| 90-day mortality ratio | -0.048 | 0.006*** |  |
| Medical interdisciplinarity | 0.270 | 0.032*** |  |
| Medical therapy offerings | 0.048 | 0.016** |  |
| Certification | 0.470 | 0.012*** |  |
| Professional recommendation (award) | 0.025 | 0.018 |  |
|  |  |  | *As % of average travel time* |
| WTT (patient recommendation) | 0.628 |  | *3.2%* |
| WTT (90-day mortality ratio) | 0.277 |  | *1.4%* |
| WTT (medical interdisciplinarity) | 0.290 |  | *1.5%* |
| WTT (certification) | 2.710 |  | *14.0%* |
| Number of patients | 66,645 |  |  |
| Number of hospitals | 862 |  |  |
| Prob>chi^2^ | 0.000 |  |  |
| Pseudo R^2^ | 0.582 |  |  |
| **Knee replacement** |  |  |  |
| *Main effects* |  |  |  |
| Travel time | -0.134 | 0.001*** |  |
| Patient recommendation | -1.071 | 0.016*** |  |
| 1-year revision rate | -0.066 | 0.004*** |  |
| Medical interdisciplinarity | 0.046 | 0.019* |  |
| Medical therapy offerings | 0.081 | 0.017*** |  |
| Certification | 0.251 | 0.009*** |  |
| Professional recommendation (award) | 0.732 | 0.012*** |  |
|  |  |  | *As % of average travel time* |
| WTT (patient recommendation) | 2.749 |  | *11.2%* |
| WTT (1-year revision rate) | 0.616 |  | *2.5%* |
| WTT (medical therapy offerings) | 0.172 |  | *0.7%* |
| WTT (certification) | 2.070 |  | *8.4%* |
| WTT (professional recommendation) | 6.035 |  | *24.6%* |
| Number of patients | 82,014 |  |  |
| Number of hospitals | 844 |  |  |
| Prob>chi^2^ | 0.000 |  |  |
| Pseudo R^2^ | 0.463 |  |  |

Notes: Conditional logit model of hospital choice for colorectal resection / knee replacement patients treated in 2017 and 2018. Coefficients represent marginal utilities. Coefficients of the covariates number of beds, hospital type, university hospital status and their interactions with patient features are not reported here but available on request along with each variable’s interactions with patient features. The hospital sample was restricted to only those hospitals that passed the data cleaning steps described in Figure 6 for both years.

*** p < 0.001; ** p < 0.01; * p < 0.05

Table A5: Estimated marginal utilities – colorectal surgery - cancer patients only

| **Colorectal resection** | Estimate | Standard Error |  |
| --- | --- | --- | --- |
| *Main effects* |  |  |  |
| Travel time | -0.194 | 0.003*** |  |
| Patient recommendation | -0.261 | 0.030*** |  |
| 90-day mortality ratio | -0.052 | 0.008*** |  |
| Medical interdisciplinarity | 0.170 | 0.044*** |  |
| Medical therapy offerings | 0.080 | 0.021*** |  |
| Procedure volume | 0.003 | 0.003*** |  |
| Certification | 0.421 | 0.421*** |  |
| Professional recommendation (award) | -0.013 | -0.013 |  |
|  |  |  | *As % of average travel time* |
| WTT (patient recommendation) | 0.442 |  | *2.3%* |
| WTT (90-day mortality ratio) | 0.299 |  | *1.6%* |
| WTT (medical therapy offerings) | 0.167 |  | *0.9%* |
| WTT (procedure volume) | 1.792 |  | *9.3%* |
| WTT (certification) | 2.425 |  | *12.6%* |
| Number of patients | 40,404 |  |  |
| Number of hospitals | 862 |  |  |
| Prob>chi^2^ | 0.000 |  |  |
| Pseudo R^2^ | 0.598 |  |  |

Notes: Conditional logit model of hospital choice for colorectal resection / knee replacement patients treated in 2017 and 2018. Coefficients represent marginal utilities. Coefficients of the covariates number of beds, hospital type, university hospital status and their interactions with patient features are not reported here but available on request along with each variable’s interactions with patient features. The hospital sample was restricted to only those hospitals that passed the data cleaning steps described in Figure 6 for both years. The patient sample was restricted to patients with cancer as their primary or secondary diagnosis.

*** p < 0.001; ** p < 0.01; * p < 0.05

Appendix A1: Detailed description of data sources

Data was obtained from the following sources:

1. Structured quality report: On a yearly base, hospitals in Germany are required to disclose structural and quality data through a structured report. Information includes besides others procedure volumes, number of beds and treatment specific process and outcome quality indicators on hospital level.
2. Patient experience questionnaires: Two leading German sickness funds cooperate with the online platform *Weisse Liste* to reach out to insurees with questionnaires to evaluate the service provided by doctors, nurses and the hospital itself. Questions are answered on a scale from 1 to 6, where 1 means the best possible and 6 the worst possible service.
3. Quality assurance with routine data program: On a yearly base, the largest German sickness fund (AOK) estimates risk-adjusted clinical outcome indicators, such as mortality rates or revision rates, for selected procedures for every German hospital.
4. Claims data on patient level: Pseudonymised claims data on patient level including choice of hospital from the AOK was made available for the purpose of this study. The patient sample comprises approximately 35% (33%) of the total procedure volume for knee replacement and 33% (35%) of the total procedure volume for colorectal resections in 2017 (2018).
5. Other sources:
   1. Medical Societies: Medical societies issue certifications to hospitals meeting certain pre-defined criteria, such as minimum volume requirements and structural quality standards.
   2. Professional recommendation: Annually, the German magazine *Focus* publishes a list of the best hospitals for each treatment area in Germany, based on hospital recommendations from inpatient physicians and structured interviews with referring outpatient physicians and specialists^[[1]](#footnote-1)^.
   3. Federal Employment Agency: The federal employment agency posts annual statistics on employment, demographics and income on their central platform.

Appendix A2: Detailed description of data cleansing process

The initial sample includes all hospitals that performed at least one procedure in 2017 and 2018 and for which all quality indicators were available. In the data cleaning process we slightly modified the initial data set to reach our final sample (*see Figure 6*):

Fig. 6 Data sample


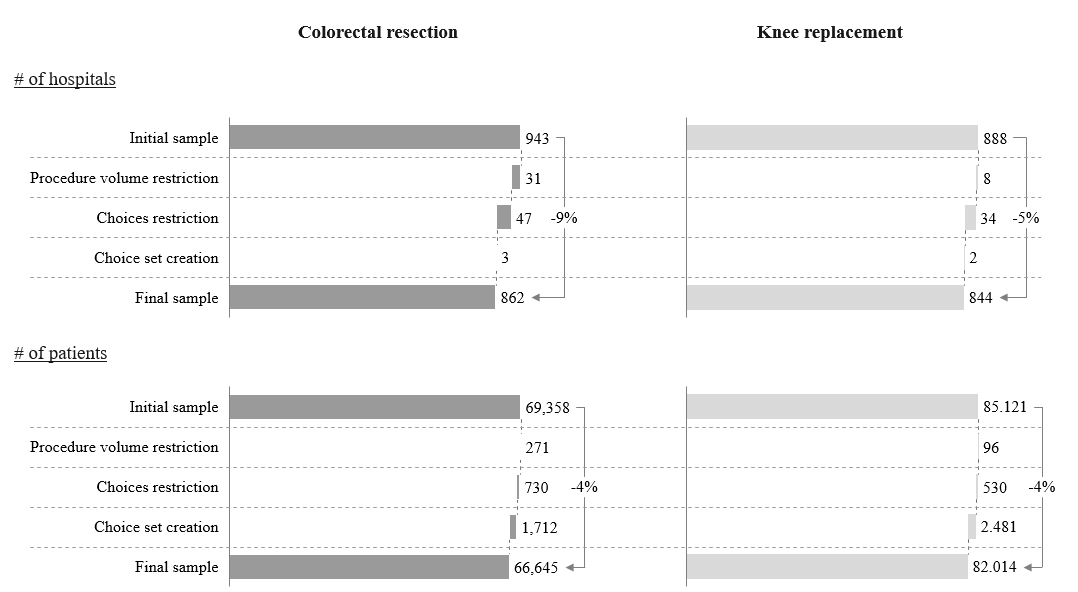
 *Notes*: *Procedure volume restriction* refers to the minimum number of cases the hospital is required to have for each treatment area per year (colorectal resection >= 15, knee replacement >=20). *Choices restriction* refers to the minimum number of patients per hospital and is set to 10 for both procedures. *Choice set creation* refers to the loss of hospitals and/or patients that were not chosen in or did not choose one of their 50^th^ nearest hospitals.

In order to minimize statistical noise and manage computational power needs of the model we filtered out all hospitals with less than 15 (20) cases for colorectal resection (knee replacement) and which were visited by fewer than 10 AOK patients in 2017 or 2018. Lastly, patients that did not choose one of their nearest 50 hospitals for treatment were also excluded from the final sample.

Fig. 7 Accessibility – comprehensibility matrix


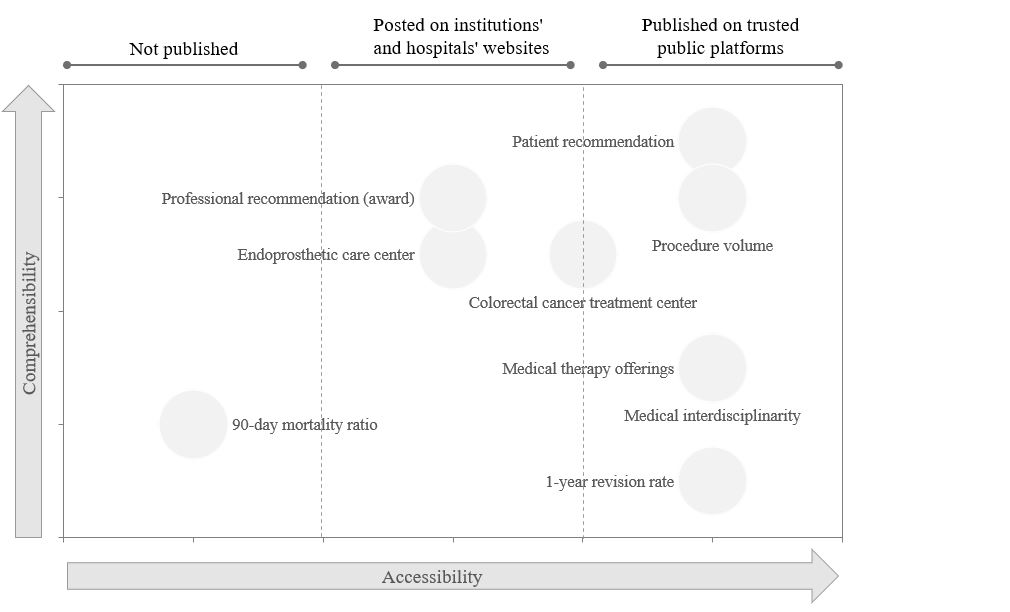


Fig. 8 Distribution of hospital choices subject to travel time - colorectal resection


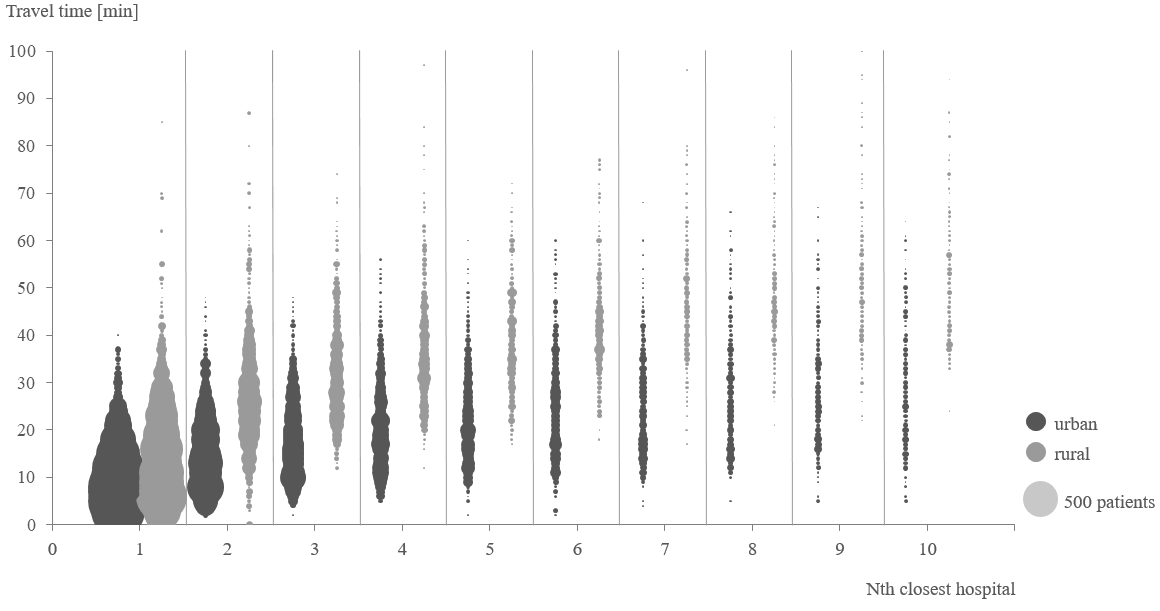


Fig. 9 Distribution of hospital choices subject to travel time - knee replacement


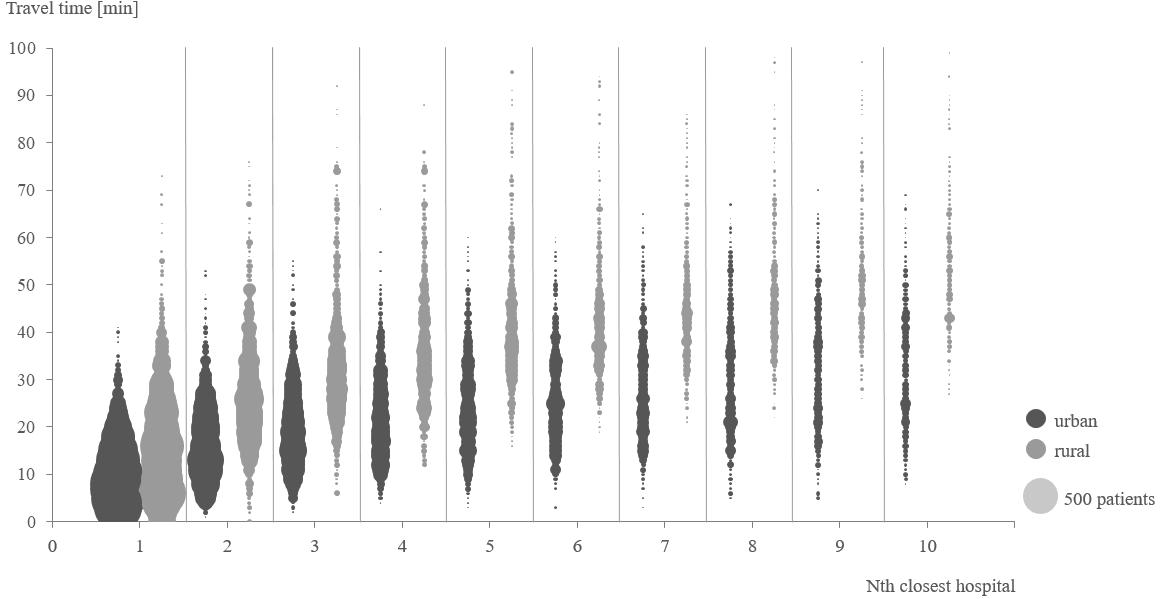


Fig. 10 Correlation matrix colorectal resection


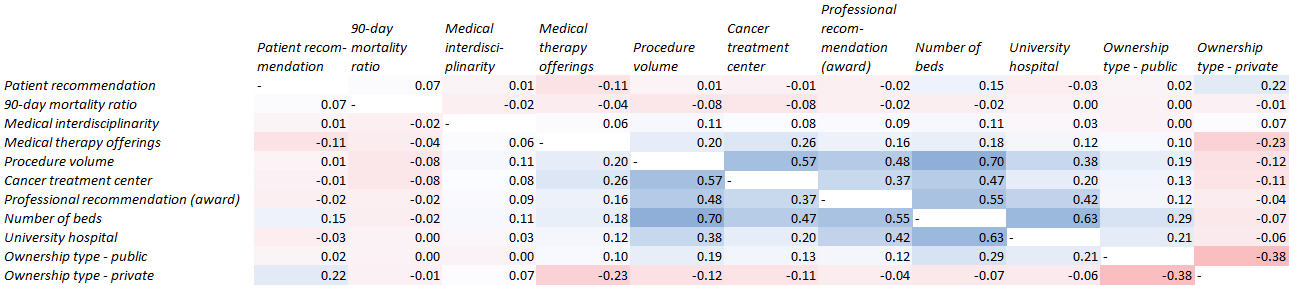


Fig. 11 Correlation matrix knee replacement


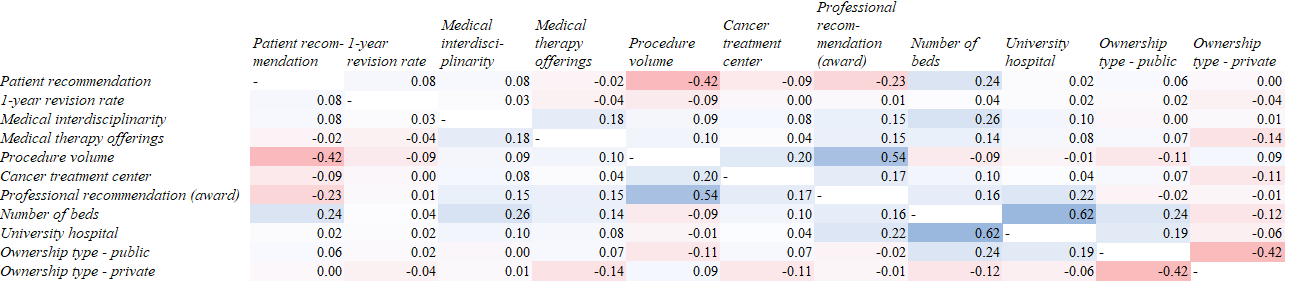


Fig. 12 Accessibility - comprehensibility matrix: Relative demand effects


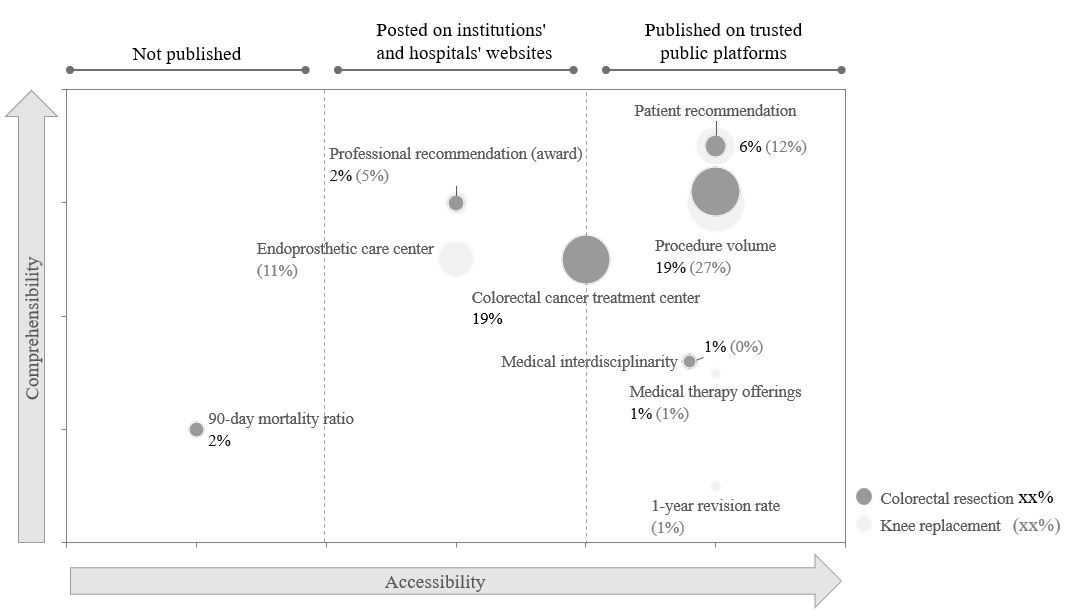


References

1. DGAV e.V. (2016) Zertifizierungsordnung Chirurgische Koloproktologie. www.dgav.de/fileadmin/media/texte_pdf/zertifizierung/zerto/Zertifizierungsordnung_Kurzform_CACP.pdf. Accessed 08 Nov 2019

2. WIdO (2016) Indikatorenhandbuch für Leistungsbereiche ohne Berichterstattung im AOK-Krankenhausnavigator: Verfahrensjahr 2016. https://www.qualitaetssicherung-mit-routinedaten.de/imperia/md/qsr/methoden/indikatorenhandbuch_2016_ohne_bericht_final.pdf. Accessed 08 Nov 2019

3. (2005) Regulations of the Federal Joint Committee pursuant to § 136b para. 1 sentence 1 no. 2 SGB V for hospitals licensed according to § 108 SGB V: Mindestmengenregelung, Mm-R

1. For more information on methodology see https://focus-arztsuche.de/ueber-uns/siegel/top-krankenhaushttps://focus-arztsuche.de/ueber-uns/siegel/top-krankenhaushttps://focus-arztsuche.de/ueber-uns/siegel/top-krankenhaus [↑](#footnote-ref-1)
